# Comparing methods to secure a tracheal tube placed via a surgical cricothyroidotomy: a randomised controlled study in cadavers

**DOI:** 10.1186/s13049-021-00925-y

**Published:** 2021-07-28

**Authors:** Christopher J Groombridge, Amit Maini, Joseph Mathew, Yesul Kim, Mark Fitzgerald, De Villiers Smit, Gerard O’Reilly

**Affiliations:** 1 National Trauma Research Institute, Melbourne, Australia; 2 Emergency & Trauma Centre, The Alfred Hospital, Melbourne, Australia; 3 School of Public Health and Preventive Medicine, Monash University, Melbourne, Australia; 4 Central Clinical School, Monash University, Melbourne, Australia

**Keywords:** Intubation, Surgical airway

## Abstract

**Objective:**

In the ‘can’t intubate can’t oxygenate’ scenario, techniques to achieve front of neck access to the airway have been described in the literature but there is a lack of guidance on the optimal method for securing the tracheal tube (TT) placed during this procedure. The aim of this study was to compare three different methods of securing a TT to prevent extubation following a surgical cricothyroidotomy.

**Methods:**

A randomised controlled trial was undertaken. The population studied were emergency physicians (EPs) attending a cadaveric airway course. The intervention was securing a TT placed via a surgical cricothyroidotomy by suture. The comparison was securing the TT using fabric tape with two different tying techniques. The primary outcome was the force required to extubate the trachea. The trial was registered with ANZCTR.org.au (ACTRN12621000320853).

**Results:**

17 emergency physicians completed intubations using all three of the securing methods on 12 cadavers for a total of 51 experiments. The mean extubation force was 6.54 KG (95 % CI 5.54–7.55) in the suture group compared with 2.28 KG (95 % CI 1.91–2.64) in the ‘Wilko tie’ group and 2.12 KG (95 % CI 1.63–2.60) in the ‘Lark’s foot tie’ group; The mean difference between the suture and fabric tie techniques was significant (*p* < 0.001).

**Conclusions:**

Following a surgical cricothyroidotomy in cadavers, EPs were able to effectively secure a TT using a suture technique, and this method was superior to tying the TT using fabric tape.

## Background

The final step in the ‘can’t intubate can’t oxygenate’ (CICO) scenario is front of neck access (FONA)[1] and whilst there are recommendations on the technique to be employed the best method of securing a standard tracheal tube (TT) placed through the cricothyroid membrane has not been clearly established.

**Fig. 1:**
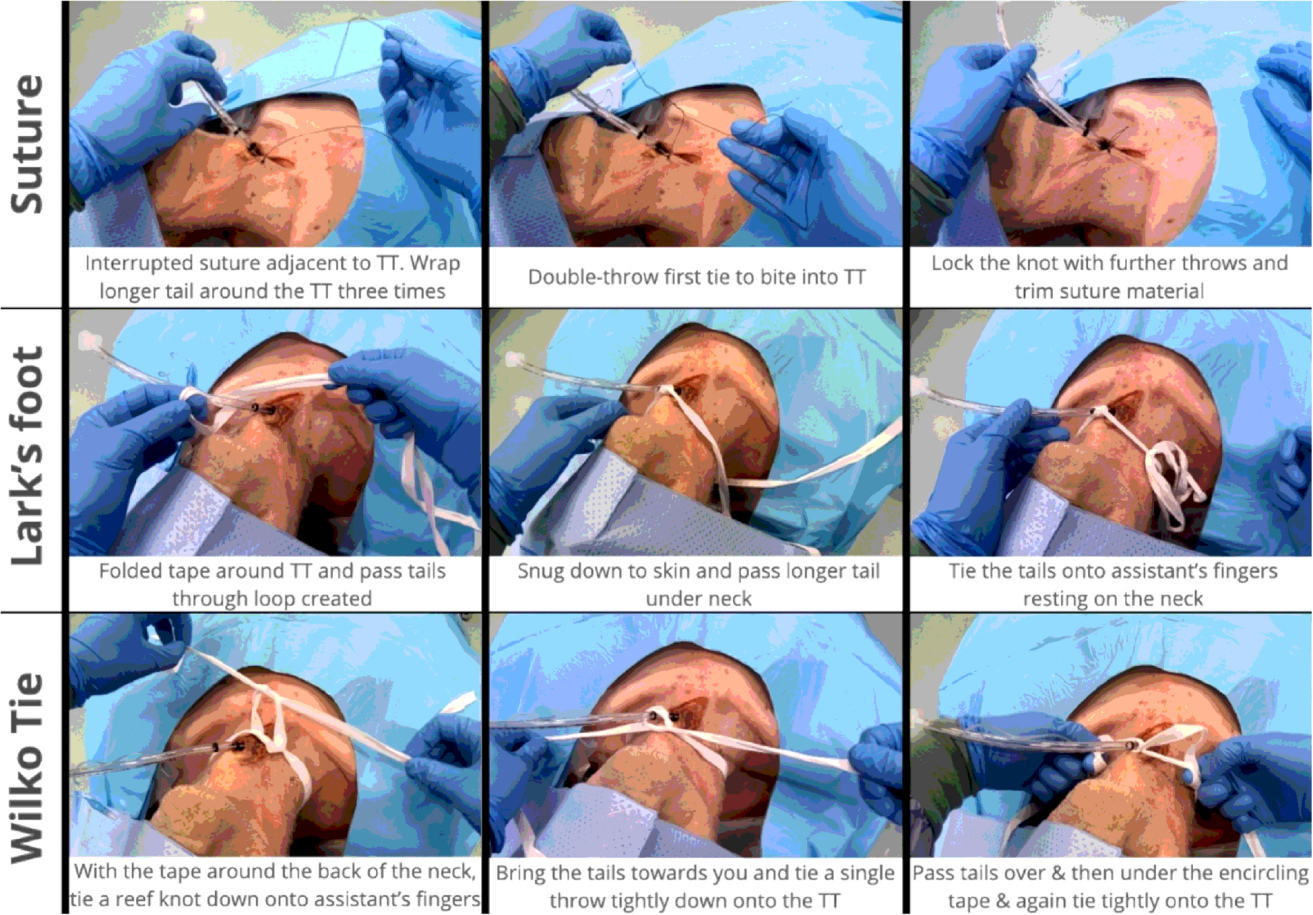
Three methods for securing the tracheal tube.

In the emergency department (ED), TTs placed during orotracheal intubation may be secured using fabric tape tied using a ‘Lark’s foot’ knot (Fig. 1)[2] or alternative knots such as the ‘the Wilko tie’ (Fig. 1). Both these methods, however, require the tape to be passed around the neck and clinicians may be reluctant to utilize these methods to secure a surgical airway, for fear of occluding venous return from the head. To avoid this risk clinicians may opt for a suture ‘drain stitch’ (Fig. 1) to maintain TT position.

Our aim is to provide clarity on an optimal method for securing a surgical airway which can be taught alongside FONA techniques. In this study we have chosen to compare the force required to extubate a fresh-frozen cadaver where the surgical cricothyroidotomy TT has been secured by either a Lark’s foot knot, using fabric tape, a ‘Wilko tie’, again using fabric tape, and a ‘drain stitch’, using a silk suture.

## Methods

### Study design and participants

This randomised controlled trial was approved by the University of Melbourne research ethics committee (approval number 1,648,354). The trial was registered before clinician enrolment at Australian New Zealand Clinical Trials Registry (ACTRN12621000320853). Twelve fresh-frozen cadavers were provided by the University of Melbourne’s department of human anatomy. Seventeen emergency physicians (EP) were recruited from The Airway Course, a one-day cadaveric airway management course for critical care clinicians.

### Intervention

Prior to the experiment, participants received instruction on the securing methods as part of The Airway Course. Each EP performed all three techniques on three different cadavers amounting to 51 experiments, the order of which was randomised in advance by computer randomisation to minimise learning effects (https://www.randomizer.org).

The TT was a Portex Choice size 6.5mm cuffed tracheal tube. The securing tape was a standard white fabric 12mm wide tape cut to 1 m lengths. The suture was a 1 Sofsilk™ 75 cm braided silk suture with a cutting needle from Covidien™.

For all the experiments the starting condition was with the supine cadaver intubated with a TT placed through a surgical airway performed as part of the course education (midline longitudinal incision, followed by horizontal cricothyroid puncture as part of a scalpel, finger, bougie technique).

After the TT was deemed secure by the participant, the cadaver was draped such that the investigator was unable to determine the securing method used.

### Outcomes

The force required to extubate the cadaver, the primary outcome, was estimated using a digital force gauge (Dr.meter digital scale ES-PS01) attached to the TT. The same investigator, blinded to the securing method, performed all measurements by applying slow gradual upwards traction on the meter, until the TT cuff was completely through the surgical wound, and recording the maximum value. Assuming Force = Mass x Acceleration, and given the slow traction technique, where acceleration was considered negligible (and consistent across experiments), the weight measurement of the gauge was taken to be a surrogate for the extubation force.

After performing each technique the participant was asked to mark a visual analogue scale (VAS) to indicate how easy or hard the technique had been to undertake following minimal instruction.

### Statistical analysis

A minimum of 10 clinicians had been determined by a power analysis based on an alpha of 0.05, a power of 80 % and a small difference in extubation force (200 g) between procedures. A sample size of 15 participants was then recruited to ensure complete data for the analysis.

Symmetrical numerical data has been summarised using the mean (SD); skewed numerical data has been summarised using the median (IQR); and categorical data has been summarised using frequencies (%). The statistical significance of these measures of association were tested using paired statistical testing procedures, i.e., Repeated Measures Analysis of Variance (ANOVA). *P* values < 0.05 were considered statistically significant.

## Results

The planned sample size was achieved with 17 clinicians completing the three techniques on 12 cadavers for a total of 51 experiments. Characteristics of the participants are presented in Table 1. The twelve cadavers had a mean height 150 cm, mean weight 66KG, and 7 were female.

**Table 1:** Characteristics of participants

Characteristic	n = 17 (%)
**Gender**	
Female	6 (35 %)
Male	11 (65 %)
**Year’s experience as EM specialist**	
< 5 years	4 (23.5 %)
5–10 years	10 (58.8 %)
11–15 years	1 (5.9 %)
> 15 years	2 (11.8 %)

EM = Emergency Medicine

**Fig. 2:**
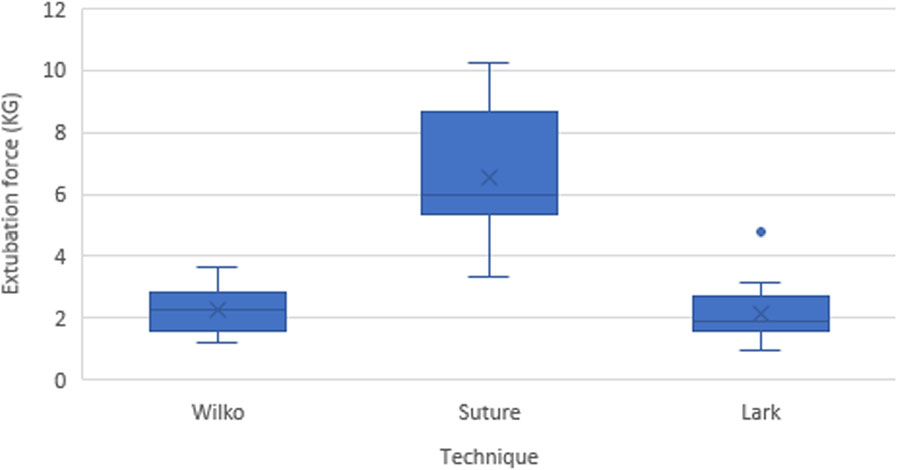
Extubation force (Kg). The boxplots show medians (solid line), means (X) and interquartile ranges (IQR). The whiskers give the range except for “outliers” that are more than ± 1.5 times the IQR.

**Fig. 3:**
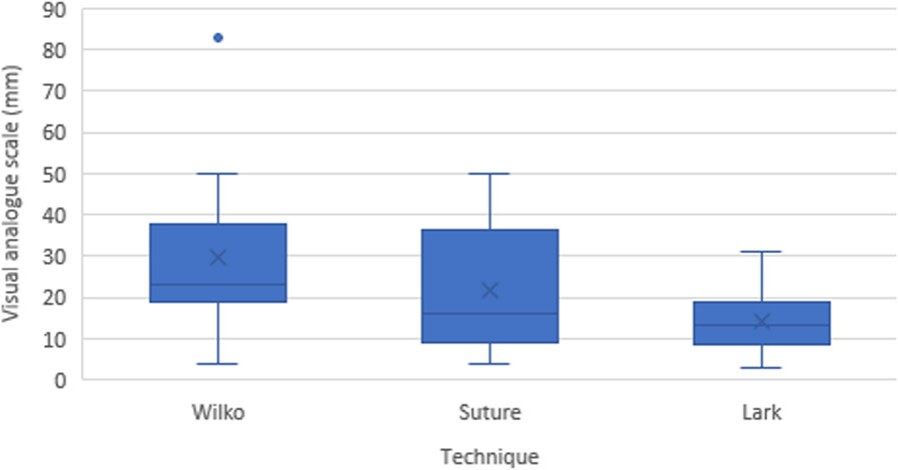
Visual Analogue Scale of technique difficulty. The boxplots show medians (solid line), means (X) and interquartile ranges (IQR). The whiskers give the range except for “outliers” that are more than ± 1.5 times the IQR.

Extubation force (Fig. 2) and VAS of technique difficulty (Fig. 3) are summarised in Table 2.

**Table 2:** Technique performance outcomes

	Lark’s foot	Wilko Tie	Suture	*p*-value
Extubation force (KG)	2.12 (1.63–2.60)	2.28 (1.91–2.64)	6.54 (5.54–7.55)	< 0.001
Visual Analogue Scale of difficulty (mm)	14.35 (9.91–18.80)	29.53 (20.10–38.96)	21.59 (13.69–29.49)	< 0.05

The mean extubation force was highest for the suture technique, followed by the Wilko Tie and then Lark’s foot technique, which had the lowest force required. The difference between these mean values was significant using a repeated measures ANOVA (*p* < 0.001), as seen in Table 2. Post-hoc pairwise comparison using a Bonferroni adjustment demonstrated a significant mean difference in force between the suture technique and the Wilko (mean difference 4.27KG; 95 % CI 3.02–5.52; *p* < 0.001) and Lark’s foot technique (mean difference 4.43KG; 95 % CI 3.44–5.42; *p* < 0.001). There was no significant difference identified between the Wilko Tie and Lark’s foot techniques.

The mean VAS was highest for the Wilko Tie technique, followed by the suture technique and then the Lark’s foot technique, which was reported as the easiest of the techniques. The difference between these mean values was significant using a repeated measures ANOVA (*p* = 0.016), as seen in Table 2. Post-hoc pairwise comparison using a Bonferroni adjustment demonstrated a significant difference between the Wilko Tie and Lark’s foot technique (mean difference 15.18mm; 95 % CI 3.42–26.94; *p* = 0.012). There was no significant difference identified between the other techniques.

## Discussion

This randomised controlled trial compared the force required to extubate a TT placed via a surgical cricothyroidotomy, using three securing techniques, and found that a suture ‘drain stitch’ method was able to resist a greater extubation force than two fabric tape tying methods. The suture technique was also not reported to be more difficult to perform than the fabric tape tie techniques.

One of the concerns with the fabric tape tie techniques is that the tape must be passed around the neck with the associated risk of occluding venous return which may affect intracranial pressure, particularly in head injured patients[3]. The technique taught in this study involved tying down onto an assistant’s three fingers, placed onto the anterolateral neck, such that when their fingers are removed the tape is not constricting the neck. This additional ‘slack’ in the system may have allowed the TT cuff to be pulled from the cricothyroidotomy and does not reflect the likely success of these tying methods to secure an orally placed TT. Prior studies have assessed different options for securing a TT placed orally, with both different types of knot[4], as well as proprietary tube holding devices[5], and adhesive tape[6, 7]. Most of these studies, however, were undertaken in mannequins and none assessed methods of securing a TT placed via surgical cricothyroidotomy.

Surgical airways are performed rarely in the ED[8] and as such may be stressful to perform. This has the potential to impair performance[9] and clear guidance on the optimal method for securing the TT after successful completion of a surgical airway should form part of education around this procedure.

We have demonstrated that EPs with a brief training intervention are able to successfully secure a TT placed via a surgical cricothyroidotomy using a silk suture, which is able to resist a mean extubation force equivalent to at least 6 KG.

There are limitations to this study. Firstly, we used cadavers rather than anaesthetised ED patients and so the results cannot be automatically extrapolated to this patient group. Fresh frozen cadavers represent the best existing alternative model for this type of research, however, particularly when compared with manikin models. Secondly, the force applied to the TT in this experiment may not be representative of accidental extubations *in vivo*, which can involve sudden application of force in any direction. Lastly, the study’s participants were a relatively experienced group of EPs who had chosen to undertake a cadaveric airway course, and this may also limit the generalisability of the findings.

## Conclusions

Following a surgical cricothyroidotomy in fresh frozen cadavers, EPs were able to effectively secure a TT using a simple suture technique and this method was superior to tying the TT using fabric tape.

## Data Availability

The anonymised datasets generated and/or analysed during the current study are available from corresponding author.
